# An Uncommon Case of Bilateral Breast Enlargement Diagnosed as Tumoral Pseudoangiomatous Stromal Hyperplasia: Imaging and Pathological Findings

**DOI:** 10.1155/2017/7603603

**Published:** 2017-12-03

**Authors:** Isabel Sollozo-Dupont, Héctor Alejandro Domínguez-Hernández, Cecilia Pavón-Hernández, Yolanda Villaseñor-Navarro, Robin Shaw-Dulin, Victor Manuel Pérez-Sánchez, Alejandro Javier España-Ferrufino, Laura Marysol Álvarez-Guadarrama, Fany Iris Porras-Reyes, M. Patricia Pérez-Badillo

**Affiliations:** ^1^Department of Radiology and Imaging, Instituto Nacional de Cancerología (INCan), Mexico City, Mexico; ^2^Department of Surgical Oncology, Instituto Nacional de Cancerología (INCan), Mexico City, Mexico; ^3^Department of Surgical Pathology, Instituto Nacional de Cancerología (INCan), Mexico City, Mexico

## Abstract

The incidence of reported pseudoangiomatous stromal hyperplasia (PASH), as well as the variability and severity of clinical presentations, is increasing in the literature. In parallel, several authors posit the need for an improved classification of PASH to avoid possible variables associated with this diagnosis. Here, we present a 25-year-old woman with PASH accompanied by severe bilateral and symmetrical breasts enlargement, highlighting an uncommon clinical presentation of PASH as much as the careful interdisciplinary review and correlation of histology and all available imaging studies to confirm the definitive diagnosis.

## 1. Introduction

Pseudoangiomatous stromal hyperplasia (PASH) is an uncommon nonneoplastic proliferation of the myofibroblastic cells in the mammary stroma [1, 2]. Clinically, PASH is frequently presented as a unilateral or bilateral focal microscopic changes. However, diffuse massive processes accompanied with masses are also reported [1, 3]. In the presence of masses, mammography typically shows well-defined masses with smooth borders [4, 5]. Meanwhile, sonographic appearances are a well-circumscribed hypoechoic or isoechoic oval masses, with enhanced through transmission [6].

To the diagnosis, core needle biopsy or vacuum-assisted biopsy is indicated in order to differentiate PASH from low-grade angiosarcoma, principally [2, 7, 8]. Histopathologic features of PASH have been clearly outlined by several authors who describe it as a complex pattern of interanastomosing empty slit-like spaces lined by spindle-shaped stromal cells that are positive for CD34, vimentin, actin, and calponin and negative for vascular markers such as factor VIII protein and CD31 [8–11]. After diagnosis, the prognosis of PASH is usually good although there are reports indicating recurrence rates ranging between 13 and 26% [12], by which the treatment of choice is generally a complete resection by wide excision [13].

From the original article by Vuitch et al. (1986) to the current literature, available data appear to be sufficient to provide an accurate diagnosis in any case of PASH. However, due to the variability in clinical features, the criteria for defining PASH, as well as the associated radiographic and pathological findings, are not well established [10, 14]. One of the points of confusion, for example, is whether PASH might be histopathologically defined as a diffuse process in the presence of masses [10]. This is important because discordance of histology and breast imaging presents a diagnostic situation that might require further evaluation, including repeating biopsy with a consequent delay in appropriate treatment [10, 13].

Here, we present a 25-year-old woman with an uncommon PASH characterized by several skin symptoms and multiple large masses, accompanied by a simultaneous and symmetrical breast enlargement. In addition, we enriched the present report discussing diagnostic criteria defining diffuse versus tumoral PASH since a discrepancy between histopathology and imaging is exposed. We suggest that this case is relevant to enrich the different clinical presentations of PASH, but also to reinforce the need for more definitive classification of PASH that helps with clinical management.

## 2. Case Report 

A 25-year-old multiparous woman presented in November 2015 with eight months' history of bilateral breast enlargement. The chief complaint was painful and swollen breasts, accompanied by multiple palpable nodules and erythema. She reported use of birth control implant by two years at the age of 23 years, after her last pregnancy.

**Figure 1:**
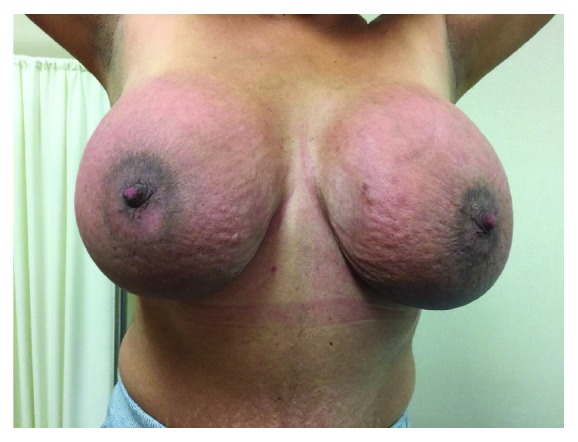
Clinical photograph of a 25-year-old woman presenting with symmetrical breast enlargement, thickened skin, and redness.

On clinical examination, the woman showed bilateral and symmetrical breast enlargement and a palpable mass of 4 cm diameter in the lower inner quadrant of the right breast, while no palpable lesions were found in the left breast. The breast skin was red and swelling, with peau d'orange appearance, whereas nipple areola complex was normal. Edema was also observed in both breasts (Figure 1). Notably, an axillary lymph node was palpable in the right axilla. At the interview, she stated no familiar or personal history of breast cancer.

**Figure 2:**
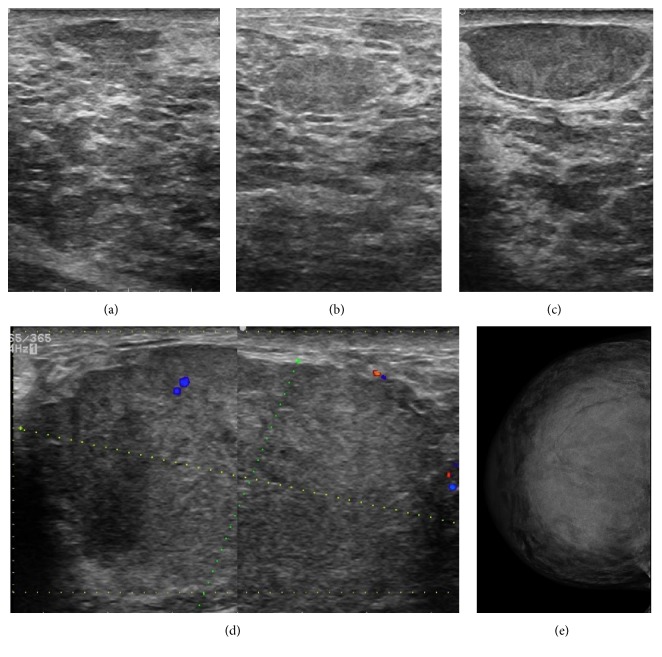
Sonographic and mammographic findings. Ultrasound of the left and right breasts showing different heterogeneous, hypoechoic, well circumscribe oval masses. Note the heterogeneous echogenicity of the breast parenchyma (a–c). Sonogram of the left breast demonstrating a hypoechoic well-circumscribed oval mass measuring 80 mm (longitudinal axis), with low flow vascularity detected by Doppler imaging (d). Craniocaudal (CC) breast mammogram of the right breast with increase in density, which decreased the sensitivity of mammography (e).

Since inflammatory breast carcinoma was suspected, a bilateral mammography was initially recommended. Nevertheless, the diagnosis was not conclusive due to the extremely dense breast tissue. Then, a complementary ultrasound was performance, revealing heterogeneous breast echotexture with hypoechoic areas and multiple circumscribed solid masses in both breasts, ranging in diameter from 3 to 8 cm (Figures 2(a)–2(e)). In addition, ultrasound examination revealed two suspicious axillary right lymph nodes with increased cortical thickness. After imaging examination, this case was categorized as BI-RADS 4A.

To the pathologic diagnostic, fragments of the large mass from the right breast were obtained by percutaneous core needle biopsy. Also, a fine needle aspiration biopsy of the right axillary lymph node was performed. Pathologists reported breast parenchyma that was composed of ductal-lobular unit and breast stromal tissue containing a complex pattern of linear spaces lined by endothelial-like spindle cells. Those cells exhibited strong positivity for CD34, actin, and calponin (Figures 4(a)–4(d)) and negativity for estrogen and progesterone receptors. The diagnosis was benign breast lump with features of a diffuse PASH. Meanwhile, fine needle aspiration biopsy of right node reported lymphoid hyperplasia.

**Figure 3:**
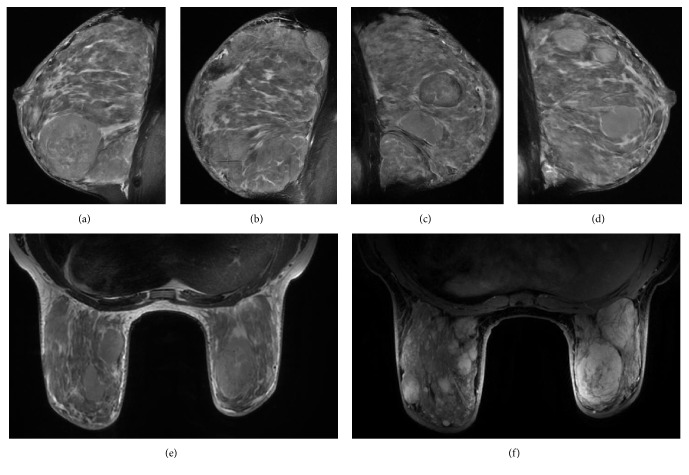
Magnetic resonance imaging findings. Right (a-b) and left (c-d) sagittal noncontrast fat suppressed T2-weighted image (T2W-FATSAT) showing extreme fibroglandular tissue and multifocal well-circumscribed masses, with internal linear reticular strands. Axial noncontrast fat suppressed T2-weighted image (T2W-FATSAT) (e) and axial contrast fat suppressed T1-weighted dynamic sequence after 6 minutes (T1WFATSAT+G) (f) demonstrating bilateral breast enlargement, with multiple circumscribed masses that present heterogeneous and persistent enhancement. Areas of nonmass enhancement with diffuse distribution also are observed in the inner and external quadrants of both breasts.

**Figure 4:**
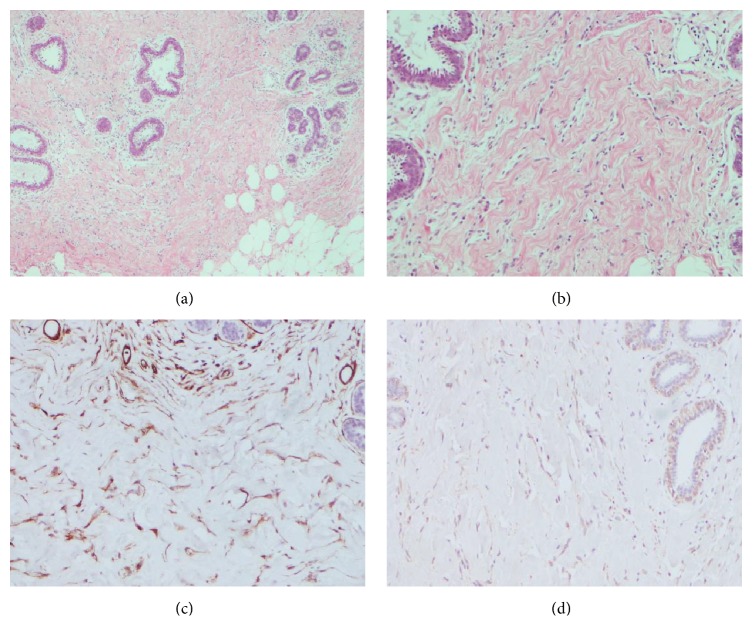
Photomicrograph shows high stromal cellularity of glandular tissue with intralobular and perilobular involvement. Slit-like spaces lined by myofibroblast (hematoxylin and eosin stain, original magnification ×10) are demonstrated (a-b), which were positive for CD34 (c) and calponin (d).

Due to the extension of the disease, the patient was subjected to a bilateral mastectomy with immediate reconstruction. A magnetic breast resonance was performed for surgical planning, confirming multiple bilateral circumscribed masses with avid and persistent enhancement after gadolinium administration, which is usually related to a benign etiology (Figures 3(a)–3(f)). After the referred surgery, and taking into account the imaging features of the lesion, the diagnosis was changed from diffuse PASH to tumoral PASH in a careful interdisciplinary review of the case.

Currently, the patient is tumor-free on one-year follow-up with excellent cosmetic outcome and breast symmetry.

## 3. Discussion

PASH of the breast is a rare benign proliferation of mesenchymal stromal cells with irregular slit-like formations resembling angiomatous structures that are similar to vascular spaces. However, these slit-like spaces are not true vascular structures and lack red blood cells. In fact, these spaces are lined by myofibroblasts that are attenuated, lack atypia, and resemble endothelial cells [8, 10, 13, 15, 16]. Although PASH is frequently incidental microscopic findings, evidence shows that its spectrum might include sizeable or symptomatic masses [10, 13, 15, 16].

In the present report, we expose a case of a 25-year-old mastectomized woman by the presence of PASH that is unique for its radiographic and clinical presentation, for example, her bilateral and pronounced symmetrical breast enlargement, accompanied by multiple large masses comprising both breasts. Despite the fact that bilateral nodular cases of PASH are reported in the English medical literature, contrary to our patient, women of those cases have been mostly presented with more growth of one of the breasts causing significant asymmetry [1, 17–19]. Also, it is common to find cases coursing with unilateral processes, or with a unique round mass that resembles a fibroadenoma or a phyllodes tumor [9, 20–23].

Notably, at the clinical examination, the woman presented with painful breasts that were hyperemic and markedly enlarged. Additionally, she had skin changes such as erythema, thickening, edema, and “peau d'orange” appearance, all of which were accompanied with increased breast hardness. The extension and severity of those symptoms led clinicians to suspect breast inflammatory carcinoma. Noteworthily, within the diagnostic possibilities, PASH was not suspected due to its relative rarity as a symptomatic disease. However, there is increasing evidence on aggressive forms of PASH, suggesting that this clinical presentation may be more common than expected [1, 16, 18, 19].

On the other hand, mammography has a limited use here because of the increased mammary density. Meanwhile, sonographic findings revealed benign-appearing, circumscribed oval hypoechoic masses ranging in size from 3 cm to 8 cm. In spite of the fact that many reports exhibit marked variability in tumoral PASH when it is evaluated through ultrasonography, this lesion is consistently defined as being oval and hypoechoic with no posterior acoustic shadowing, varying in size between 1 cm and 12 cm [3, 5, 24, 25], which concur with our breast imaging.

In addition, ultrasonographic features of PASH were corroborated by using magnetic resonance imaging (MRI), where circumscribed masses with isointense signal as that of the surrounding parenchyma on T1 weighted images were revealed. The enhancement was type 1, corroborating the benign etiology of the disease. Nonetheless, there are reports indicating that curves generated for dynamic contrast also can be of type II [24, 26]. Even though nonspecific markers of PASH were defined by using MRI, we consider that the present findings are important because of more recent reports describing mammographic and ultrasonographic features of PASH [16]. However, little is known about the MRI characteristics up to today [24, 26].

Multimodality imaging patterns of PASH presented here are suggestive but not conclusive. Thus, following BI-RADS criteria, the initial recommendation was core needle biopsy by which histopathological examination revealed a “diffuse PASH.” Noteworthily, this diagnosis was originally focused on stromal changes and immunohistochemistry. However, following an interdisciplinary review of this case, the diagnosis changes from “diffuse” to “tumoral PASH,” an aspect that was supported by imaging.

Undoubtedly, the aggressive form of stromal hyperplasia exposed here is not similar to what is reported in the literature. This is demonstrated by the quality of imaging and pathologic representation suggesting that this case might contribute to the differential diagnosis of a rapidly growing breast mass.

On the other hand, some authors assert that, under today's literature, it is no longer possible to obtain an assertive diagnosis of PASH due to inconsistences underlying its classification, an aspect that could be inadequate for the patient management after the initial biopsy [10]. One of the points of confusion, for which this report argues, is whether PASH could be pathologically defined as a diffuse process when it results in a mass lesion by imaging [10, 24, 27]. Although, with one case, the exposed confusion will never be resolved, according to previous reports [10], we consider that a diffuse PASH in the presence of masses is technically concordant with the diagnosis of “tumoral PASH.”

Concerning etiology, the most prevalent theory is that of a hormone-dependent process on the basis of observations that PASH is most frequently seen in premenopausal women or in elderly women taking hormone-replacement therapy [2, 10]. Therefore, it is possible to suggest that the use of contraceptive in an altered hormonal milieu could modify the reactivity of myofibroblasts causing the masses-forming PASH in our patient [10, 11, 25, 27, 28]. Lastly, because of the extension of the disease and severity of the clinical presentation, the treatment of choice was bilateral subcutaneous mastectomy with immediate silicone implant reconstruction, producing a satisfactory cosmetic result.

In conclusion, we present a unique case of a PASH that demanded an aggressive treatment due to its extended presentation. With the apparent discrepancy present here, we attempt to emphasize that it is not enough to check only pathology, but also the sequence of actions underlying the diagnosis should be checked, including clinical findings and imaging modalities that may be needed to ensure correct diagnosis.
